# Comparative Genomics of *Xanthomonas euroxanthea* and *Xanthomonas arboricola* pv. *juglandis* Strains Isolated from a Single Walnut Host Tree

**DOI:** 10.3390/microorganisms9030624

**Published:** 2021-03-17

**Authors:** Camila Fernandes, Leonor Martins, Miguel Teixeira, Jochen Blom, Joël F. Pothier, Nuno A. Fonseca, Fernando Tavares

**Affiliations:** 1 CIBIO—Centro de Investigação em Biodiversidade e Recursos Genéticos, InBIO-Laboratório Associado, Universidade do Porto, Rua Padre Armando Quintas 7, 4485-661 Vairão, Portugal; leonor.martins@cibio.up.pt (L.M.); mamagalhaesteixeira@gmail.com (M.T.); nuno.fonseca@cibio.up.pt (N.A.F.); 2 FCUP—Departamento de Biologia, Faculdade de Ciências, Universidade do Porto, Rua do Campo Alegre, 4169-007 Porto, Portugal; 3 Unidade Estratégica de Investigação e Serviços de Sistemas Agrários e Florestais e Sanidade Vegetal, INIAV, Avenida da República, Quinta do Marquês, 2780-157 Oeiras, Portugal; 4 Bioinformatics and Systems Biology, Justus-Liebig University Giessen, Ludwigstraße 23, 35390 Giessen, Germany; jochen.blom@computational.bio.uni-giessen.de; 5 Environmental Genomics and Systems Biology Research Group, Institute for Natural Resource Sciences, Zurich University of Applied Sciences (ZHAW), Einsiedlerstrasse 31, 8820 Wädenswil, Switzerland; joel.pothier@zhaw.ch

**Keywords:** walnut bacterial blight, comparative genomics, *Xanthomonas arboricola* pv. *juglandis*, *Xanthomonas euroxanthea*, pathogenicity

## Abstract

The recent report of distinct *Xanthomonas* lineages of *Xanthomonas arboricola* pv. *juglandis* and *Xanthomonas euroxanthea* within the same walnut tree revealed that this consortium of walnut-associated *Xanthomonas* includes both pathogenic and nonpathogenic strains. As the implications of this co-colonization are still poorly understood, in order to unveil niche-specific adaptations, the genomes of three *X. euroxanthea* strains (CPBF 367, CPBF 424^T^, and CPBF 426) and of an *X. arboricola* pv. *juglandis* strain (CPBF 427) isolated from a single walnut tree in Loures (Portugal) were sequenced with two different technologies, Illumina and Nanopore, to provide consistent single scaffold chromosomal sequences. General genomic features showed that CPBF 427 has a genome similar to other *X. arboricola* pv. *juglandis* strains, regarding its size, number, and content of CDSs, while *X. euroxanthea* strains show a reduction regarding these features comparatively to *X. arboricola* pv. *juglandis* strains. Whole genome comparisons revealed remarkable genomic differences between *X. arboricola* pv. *juglandis* and *X. euroxanthea* strains, which translates into different pathogenicity and virulence features, namely regarding type 3 secretion system and its effectors and other secretory systems, chemotaxis-related proteins, and extracellular enzymes. Altogether, the distinct genomic repertoire of *X. euroxanthea* may be particularly useful to address pathogenicity emergence and evolution in walnut-associated *Xanthomonas*.

## 1. Introduction

*Xanthomonas* is a genus of gammaproteobacteria [1], which include numerous species acknowledged as important plant-associated bacteria with the capacity to cause disease in a wide range of plant species, including important agricultural crops [2,3]. At the current date, the genus comprises 32 species (with validly published and correct names) [4], some of which are subdivided into distinct pathogenicity groups, also known as pathovars, according to their high degree of host specificity, disease symptoms, and infection mechanisms [5,6,7,8]. Within this genus, *Xanthomonas arboricola* [9] is responsible for severe diseases in important stone fruits and nut trees [10]. Pathogenic strains of *X. arboricola* belonging to the pathovar *juglandis* [11] cause serious diseases on walnut, namely walnut bacterial blight (WBB), brown apical necrosis (BAN), and vertical oozing canker (VOC), altogether responsible for major drops in walnut production and extended damages in walnut nurseries, leading to major economic losses [12,13,14].

Compared to other *X. arboricola* pathovars, *X. arboricola* pv. *juglandis* is characterized by a larger genetic diversity recorded throughout geographically distinct walnut cultivation regions [15,16,17,18,19]. Genotyping and population studies of walnut-associated xanthomonads identified a high diversity of *X. arboricola* pv. *juglandis* pathogenic strains responsible for walnut bacterial diseases [13] and nonpathogenic strains of *X. arboricola* shown to be asymptomatic on walnut [20]. Based on a large body of evidence, carefully reviewed by Büttner and Bonas (2010) [21] and more recently by An et al. (2020) [22], it is currently acknowledged that *Xanthomonas* pathogenesis is dependent on an array of pathogenicity and virulence factors, including type 3 secretion system (T3SS) and type 3 effectors (T3E), but also other type secretion systems, adhesins, extracellular polysaccharides (EPS), lipopolysaccharides (LPS), and plant cell wall hydrolytic enzymes, among others. Furthermore, it has been suggested that these genetic determinants of pathogenicity and virulence are under the control of tight transcriptional and post-transcriptional regulatory mechanisms that allow infective bacteria to adhere, invade the plant host tissues, and to multiply and overcome the plant defense mechanisms [21].

Comparative genomics of pathogenic and nonpathogenic strains of *X. arboricola* has been instrumental to unveil genetic determinants of pathogenicity and virulence [23,24,25] and to provide insights into evolutionary events linked to pathoadaptation, as emphasized by Cesbron et al. (2015) [24]. Still, in order to gain more insight regarding adaptations of nonpathogenic strains to new environments, comparative genomics of pathogenic and nonpathogenic strains isolated from the same host is required [22]. Although T3SS and T3E have been pointed out as major players of pathogenicity in *X. arboricola*, the different repertoires of virulence-associated genes between pathogenic and nonpathogenic strains of *X. arboricola* suggests that pathogenicity and virulence are determined by a complex network of genetic determinants, which is not fully understood [24]. 

Most of the *X. arboricola* whole genomes currently available are scattered in multiple contigs assembled from millions of short-reads (typically < 300 bp) obtained by sequencing-by-synthesis technologies (such as Illumina) incapable of resolving repetitive domains and impairing accurate comparisons across wide genomic domains [26]. More recently, single-molecule-sequencing technologies (such as Oxford Nanopore Technologies (ONT)) have emerged. These, are capable of producing long reads (up to several thousand bp) that may span over entire repetitive regions, achieving a complete structural resolution of genetic elements, regardless the higher error rates (above 10% of mismatches and indels, compared to ~1% of mismatches for short reads) that blur the sequence resolution [26,27]. Hybrid assembling approaches, capable to conciliate the accuracy of Illumina short-reads and the gap-free long-read sequences obtained by ONT, result in single chromosome scaffolds with high confidence per base call, and particularly suitable for high accuracy comparative genomics to unveil genetic and structural variants [28,29].

The recent description of pathogenic and nonpathogenic strains isolated from walnut belonging to the new species *Xanthomonas euroxanthea* [30] raises further questions regarding new pathoadaptations to this host [19,31,32]. Furthermore, the fact that these *X. euroxanthea* strains were isolated from the same walnut host tree, together with characteristic *X. arboricola* pv. *juglandis* strains (CPBF 427, CPBF 1521) [19,30,33,34], suggest a sympatric lifestyle that may contribute to unveil events of genetic recombination and trade-offs related to pathogenicity and virulence of *Xanthomonas* in walnut. The present work discloses a comprehensive comparative genomics study between the complete genome sequences of three *X. euroxanthea* strains (CPBF 367, CPBF 424^T^, CPBF 426) and one *X. arboricola* pv. *juglandis* strain (CPBF 427) isolated from the same diseased walnut tree. To better understand the ecology and evolution of these two walnut-associated *Xanthomonas* species, emphasis has been given to the genetic determinants of pathogenicity and virulence and putative niche-specific adaptations.

## 2. Materials and Methods

### 2.1. Xanthomonas Strains Used in This Study and Genome Sequencing

Four isolates (CPBF 367, CPBF 424^T^, CPBF 426, and CPBF 427) obtained from a single walnut tree host located in the region of Loures, Portugal [19], with distinct genotypes and pathogenicity in walnut (while CPBF 367 was nonpathogenic, CPBF 424^T^ and CPBF 427 were pathogenic) [30], were chosen for genome sequencing. A previous MLSA of *acnB, fyuA, gyrB*, and *rpoD* genes grouped the strains CPBF 427 along with other *X. arboricola* pv. *juglandis* strains [19], whereas CPBF 367, CPBF 424^T^, and CPBF 426 were recently described as belonging to the new species *X. euroxanthea* [30].

Bacteria culture, DNA extraction for sequencing, library preparation, and genome sequencing and annotation were carried out as previously described by Teixeira et al. [32,34]. Briefly, bacteria were recovered on culture medium M2 at 28 °C and 100 rpm for 48 h. DNA was extracted using an E.Z.N.A. bacterial DNA purification kit (Omega Bio-tek, Norcross, GA, USA) and sequenced with Illumina and Oxford Nanopore Technologies (ONT) MinION platforms. Illumina sequencing was outsourced to GATC Biotech, AG (Konstanz, Germany), using an Illumina HiSeq instrument with a standard 2 × 150 bp paired-end library protocol, while ONT sequencing was performed on a MinION sequencer using an R9.4.1 flow cell. Reads were base called and demultiplexed using Guppy v3.4.1 (high accuracy base-calling mode) and assembled de novo following a hybrid Nanopore-Illumina approach using Unicycler v. 0.4.8 [35] and annotated with PGAP v2020-03-30.build4489 [36]. The complete genome sequences of *Xanthomonas* strains CPBF 367, CPBF 424^T^, CPBF 426, and CPBF 427 have been deposited in the European Nucleotide Archive (ENA). Illumina reads, ONT reads, and the assembled genomes are respectively available under the following accession numbers: ERX2780809, ERX4296808, and GCA_903989455 for CPBF 367; ERR2767968, ERX4911540, and GCA_905187425 for CPBF 424^T^; ERX2780811, ERX4296809, and GCA_903989465 for CPBF 426; and ERX2780812, ERX4296810, and GCA_903989475 for CPBF 427.

### 2.2. Average Nucleotide Identity

The average nucleotide identity (ANI), based on BLASTn, was carried out with OrthoANI v1.40 [37] to determine the genetic distance between each of the four bacterial genomes (CPBF 367, CPBF 424^T^, CPBF 426, and CPBF 427) sequenced within the framework of this study and 40 genomes of *Xanthomonas* spp., including 29 genomes of *X. arboricola* and eight distinct pathovars (Table S1), all available at the NCBI genome database. 

### 2.3. Comparative Genome Analysis

A total of 1149 genes from BUSCO (v. 4.0.6, database xanthomonadales_odb10) [38,39], present in all the 44 mentioned *Xanthomonas* genomes, were aligned with TCoffee (v11.00.8cbe486, mcoffee mode) [40] to generate a tree, using RAxML (raxmlGUI 2.0, model GTR+FO) [41,42] with 500 bootstrap replicates. The corresponding phylogram was visually represented with the R package “ggtree” [43]. In order to unveil strain-specific features and pools of genes shared between the CPBF 367, CPBF 424^T^, CPBF 426, and CPBF 427, a pairwise comparison of genomes based on SRVs [44,45] was performed in the EDGAR platform (EDGAR v. 2.3. [45,46] and represented in a Venn diagram.

### 2.4. Homologous of Pathogenicity and Virulence-Associated Proteins Inferred by tBLASTn Analysis

The genome sequences of the four *Xanthomonas* strains used in this study were scrutinized for the presence of protein homologs by tBLASTn analysis (tBLASTn v. 2.10.1 [47]) against a created database of protein sequences previously reported to be involved in the pathogenesis and virulence of *Xanthomonas* (Table S3). To ensure that only closely-related orthologous proteins were selected, the tBLASTn cut-offs criteria to identify protein homologs were ≥ 40% identity and ≥ 75% query sequence length. The search for homologs was performed using as query sequences proteins of xanthan biosynthesis identified by Lee et al. [48] and Vorhölter et al. [49], a list of proteins of the flagellar system from *X. campestris* pv. *vesicatoria* 85-10 [50] and from *X. fragariae* LMG 25863 (AJRZ00000000.1, NCBI database), as well as proteins of the *rpf* gene cluster for regulation of pathogenicity factors in *X. campestris*, [51] and *X. fragariae* LMG 25863, AJRZ00000000.1, NCBI database). Homologs of chemotaxis and methyl-accepting chemotaxis proteins, proteins involved in the biosynthesis of quorum sensing signals and non-fimbrial adhesins, were identified using as query the protein sequences previously used for *X. arboricola* genomes by Garita-Cambronero et al. [25]. The presence or absence of homologs associated with components of the different secretion systems were also predicted, using as query sequences proteins of the type II secretion system (T2SS) [52,53] and related hemicellulolytic, cellulolytic, and pectolytic enzymes, lipases, and proteases [25]; proteins of the type IV secretion system and type IV pilus [25,54]; and proteins of the type VI secretion system [55].

## 3. Results

### 3.1. General Features of X. euroxanthea and X. arboricola pv. juglandis Genome Assemblies

The complete genome sequences obtained by hybrid assemblies of Illumina and Nanopore reads for the four studied strains (CPBF 367, CPBF 424^T^, CPBF 426, and CPBF 427), allowed a single chromosomal scaffold to be achieved for all four strains and revealed the presence of one plasmid in strains CPBF 367 and CPBF 426 (Table 1). When comparing the genome properties, while some features are similar between the four genomes, namely a G + C content around 65%, a number of coding genes proportional to genome size, and the number of rRNA operons and 5S tRNA genes, some other features are clearly distinct between the three *X. euroxanthea* strains (CPBF 367, CPBF 424^T^, and CPBF 426) and the *X. arboricola* pv. *juglandis* strain CPBF 427. These differences are particularly underlined by a higher genome size for the *X. arboricola* pv. *juglandis* strain CPBF 427 (5.23 Mb), in comparison with the slightly smaller genomes of *X. euroxanthea* strains CPBF 367, CPBF 424^T^, and CPBF 426, i.e., less than 5.0 Mb, the higher number of RNA genes, non-coding RNAs (ncRNA), and pseudogenes observed for strain CPBF 427 comparatively with the three *X. euroxanthea* strains (Table 1).

### 3.2. Genomic Distance Assessed by ANI and Phylogenetic Analysis 

**Figure 1:**
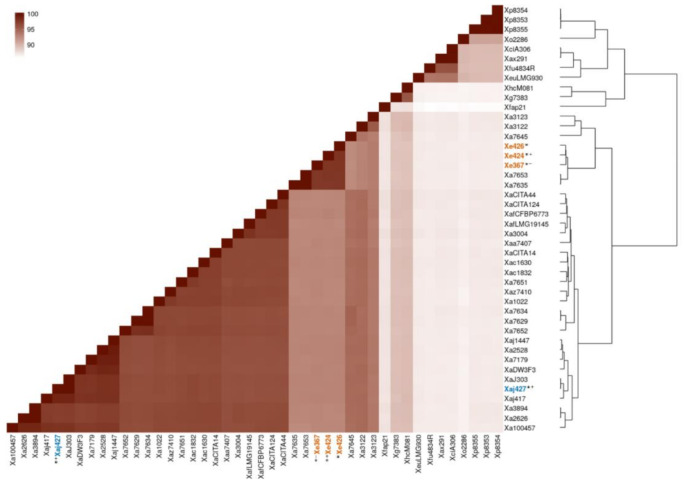
Heatmap representing average nucleotide identity (ANI) (disclosed in Table S2) and the respective distance tree cladogram, determined for 44 *Xanthomonas* genomes including the four genomes disclosed in this study, indicated with (*) and highlighted in orange (CPBF 367, CPBF 424^T^, CPBF 426) or blue (CPBF 427). Pathogenic and nonpathogenic on walnut are marked with (+) or (−), respectively. The color scale ranging from white to dark red depicts lower to higher similarity values, respectively. The strain names refer to the code field from Table S1.

**Figure 2:**
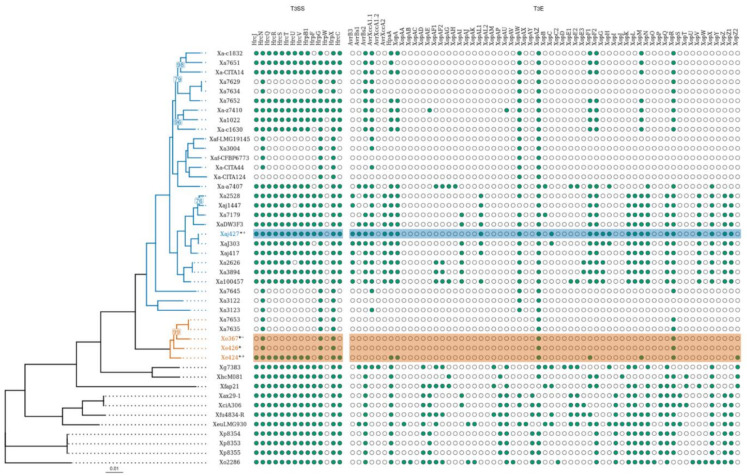
Maximum likelihood tree based on concatenated sequences of the 1149 core genes of 44 *Xanthomonas* genomes, including the *Xanthomonas euroxanthea* strains (branches in orange) and *Xanthomonas arboricola* strains (branches in blue). Phylogenetic relations were inferred using RaxML and the phylogram represented with the R package “ggtree”. Supporting values from 500 bootstrap replicates are indicated near nodes. Dot plot scheme represents the presence/absence scheme for type 3 secretion system (T3SS) and effectors (T3E) putative homologs; ●, present; ○, not present; considering a tBLASTn hit with a query coverage threshold ≥ 75%, and sequence identity with ≥ 40% cut-off. Results for genomes disclosed in this study are marked with (*); also, *X. euroxanthea* strains CPBF 367, CPBF 424^T^, and CPBF 426 are highlighted in orange and for *X. arboricola* pv. *juglandis* CPBF 427 in blue. Pathogenic and nonpathogenic on walnut are marked with (+) or (−), respectively. The strain names refer to the code field from Table S1. Best BLAST results and accession numbers of sequences used as query are disclosed in Table S3.

The ANI values determined for a sampling of 44 *Xanthomonas* strains, including the four walnut-associated *Xanthomonas* isolates characterized in the current study, assigned strains CPBF 367, CPBF 424^T^, and CPBF 426 to the recently described new species *X. euroxanthea* [30], and strain CPBF 427 as a member of *X. arboricola* pv. *juglandis*. In fact, the high ANI similarity values of ≥ 97.9% shared between strains CPBF 367, CPBF 424^T^, and CPBF 426 and the ANI values of ≤ 93.6 and ≤ 89.4% observed between these three strains and *X. arboricola* and CPBF 427 or other *Xanthomonas* species strains, respectively (Figure 1, Table S2), are clearly above and below the high stringent threshold of > 95% to separate different species [56]. Interestingly, two nonpathogenic *X. arboricola* strains (CFBP 7635 and CFBP 7653), previously described [20], share ANI values of ≥ 97.8% with the three *X. euroxanthea* strains (CPBF 367, CPBF 424^T^, and CPBF 426), strongly suggesting that these two strains are misclassified and likely belong to *X. euroxanthea*. The phylogenetic tree obtained for the *Xanthomonas* 44 strains using the 1149 single-copy orthologous genes from BUSCO placed *X. euroxanthea* strains (CPBF 367, CPBF 424^T^, and CPBF 426) and CFBP 7635 and CFBP 7653 in a cluster well separated from all the other xanthomonads considered in the analysis, including the CPBF 427 and other *X. arboricola* pv. *juglandis* strains with which they share the same plant host (Figure 2).

### 3.3. Genetic Patrimony Retrieved from the X. euroxanthea and X. arboricola pv. juglandis Strains Isolated from a Single Walnut Host Tree

**Figure 3:**
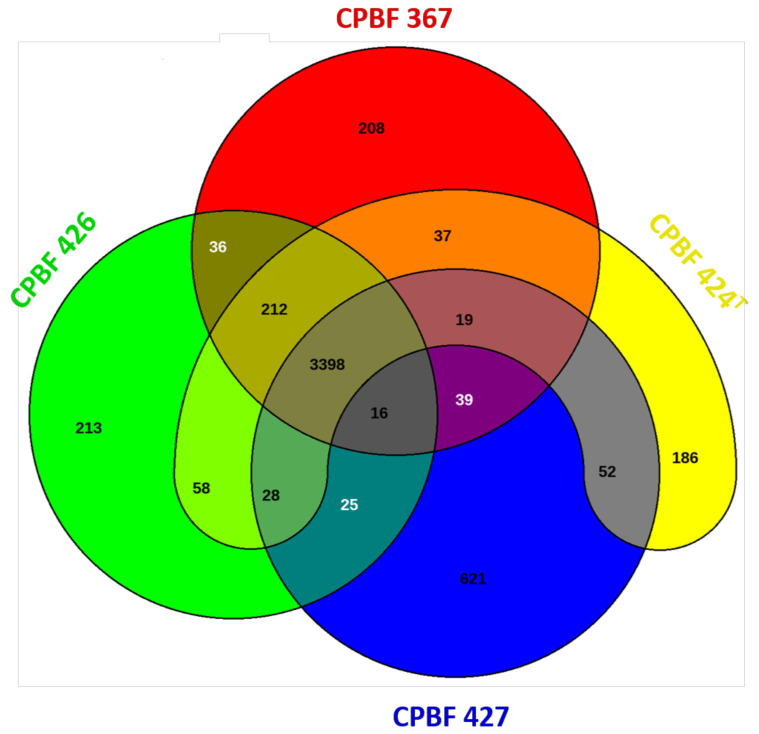
Venn diagram highlighting the genomic patrimony from CPBF 367, CPBF 424^T^, CPBF 426, and CPBF 427 genomes. The core genome is given by the interception of the four strains and corresponds to the number of orthologous CDSs shared (3398 CDSs). Strain specific CDSs are also represented in the periphery of the diagram (208 for CPBF 367, 213 for CPBF 426, 186 for CPBF 424^T^, and 621 for CPBF 427). The remaining combinations represent the number of orthologous shared between two to three genomes. The strain names refer to the code field from Table S1.

To disclose the gene pool of xanthomonads found in a single walnut tree host, the total number of CDSs corresponding to non-redundant genes retrieved from the four strains studied (CPBF 367, CPBF 424^T^, CPBF 426, and CPBF 427) was determined. The results highlighted in a Venn diagram showed that the core genome, i.e., the set of genes common to the four strains (Figure 3). The remaining CDSs, which corresponds to the accessory genome, represent the differential gene content of these strains; that is, they include the strain-specific CDSs and genes shared by two or more strains. When focusing on genomic sub-sets, 212 CDSs are shared exclusively by *X. euroxanthea* strains (CPBF 367, CPBF 424^T^, and CPBF 426), and 52 CDSs are shared exclusively by the two pathogenic strains (CPBF 424^T^, and CPBF 427). Among the genes shared by *X. euroxanthea*, a great number of regulator proteins and hypothetical proteins were found, with the latter constituting 24% of the total genes shared by *X. euroxanthea*. Regarding strain-specific genomic contents, 621 strain-specific CDSs were retrieved for *X. arboricola* pv. *juglandis*, while for *X. euroxanthea* strains, 208 unique CDSs were identified for CPBF 367, 213 unique CDSs for CPBF 426, and 186 unique CDSs for CPBF 424^T^. From these singletons, 60%, 52%, and 45% were assigned as hypothetical proteins for CPBF 367, CPBF 424^T^, and CPBF 426, respectively.

### 3.4. Pathogenic and Virulence-Related Factor Prediction

In addition to the functional analysis, the profile of *Xanthomonas* pathogenicity and virulence factors characterized in previous studies was determined, unravelling notable differences between the consortium strains isolated from the same walnut host (Figures S1–S6, Table S3). All four xanthomonad strains were found to share numerous genes associated with pathogenesis, namely genes for the biosynthesis of the xanthan polysaccharide biosynthesis (operon *gumBCDEFGHIJKLMN*), lipopolysaccharide biosynthesis, the flagellar system, the regulatory rpf cluster of pathogenicity factor synthesis, the *xps* gene of T2SS (*xpsD*, *E*, *F*, *G*, *H*, *I*, *J*, *K*, *L*, and *M*), homologs of the type IV pilus (T4P), several T4SS genes (*virB1*, *virB2*, *virB3*, *virB4*, *virB6*, *virB8, virB9, virB10, virB11*, and *virD4*), as well as genes from the T6SS (Table S3). Regarding the T2SS, the *xpsN* gene was only present in the *X. euroxanthea* strains (CPBF 367, CPBF 424^T^, and CPBF 426) (Table 2, Figure S1), and five T4P-related genes (*pilY1*, *pilX, pilW, pilV*, and *fimT*) were found to be exclusively present in the pathogenic CPBF 424^T^ (Table 2, Figure S2). Concerning the presence of non-fimbrial adhesins, it is noticeable that the *X. euroxanthea* CPBF 424^T^ and CPBF 426 strains did not harbor homologous genes for *fhaB1* and *fhaB2*, which encode filamentous hemagglutinin-related proteins, regardless of the fact that all four strains share at least three homologs to non-fimbrial adhesins (Table 2, Figure S3). Additionally, no major differences were observed between the four strains studied regarding genes encoding proteins associated with *Xanthomonas* sensing and chemotaxis mechanisms, with the exception of a methyl-accepting chemotaxis protein and a chemotaxis protein of which homologs were present in all three *X. euroxanthea* strains but not in *X. arboricola* pv. *juglandis* strain CPBF 427 (Table 2, Figure S4). The main differences between *X. arboricola* pv. *juglandis* and *X. euroxanthea* strains were observed in the profile of pectolytic enzymes associated with T2SS (Table 2, Figure S5). Homologs for a pectate lyase E and a pectinesterase were only identified in the genomes of the three *X. euroxanthea* strains. On the contrary, homologs of a degenerated pectate lyase, an endoglucanase, a rhamnogalacturonase B, and a polygalacturonase were present in CPBF 427 and not in *X. euroxanthea* strains. Furthermore, homologs of xylosidase/arabinosidase (*xylB*) were found in the two *X. arboricola* pv. *juglandis* strains CPBF 427 and in the pathogenic *X. euroxanthea* strain CPBF 424^T^.

### 3.5. Type 3 Secretion System and Its Effectors

The presence of homologous proteins for the type 3 secretion system and its effectors were evaluated in CPBF 367, CPBF 424^T^, CPBF 426, CPBF 427, and other *Xanthomonas* genomes, including known pathogenic and nonpathogenic *X. arboricola* strains (Figure 2 and Figure S6). For both T3SS and T3E proteins, *X. euroxanthea* CPBF 367 and CPBF 426 presented the same profiles as nonpathogenic *X. arboricola* strains CFBP 7653 and CFBP 7635 and a reduced number of homologs than pathogenic *X. euroxanthea* strains CPBF 424^T^. Particularly for T3SS, *X. euroxanthea* strains CPBF 367 and CPBF 426 revealed a similar profile to nonpathogenic CFBP 7653, CFBP 7635, CFBP 7645, CFBP 7634, CFBP 7629, CITA 124, and CITA 44 with the presence of only HrpG, HrpX, and HrcN. Conversely, pathogenic *X. euroxanthea* strain CPBF 424^T^ demonstrated a more complete profile that includes HrpG, HrpX, HrcN, HrcJ, HrcQ, HrcR, HrcS, HrcT, HrcV, HrpB1, HrcC, and HrcU, similarly to *X. arboricola* pathogenic strains including CPBF 427 (strain isolated from the same walnut tree host, at the same sampling event) and nonpathogenic *X. arboricola* strains CFBP 7652, CFBP 7651, and CITA 14 (Figure 2 and Figure S6). Regardless of the arsenal of T3SS proteins observed for CPBF 424^T^, this strain includes fewer T3 effector proteins (XopAZ, XopR, HpaA, XopM, XopF1, XopA, XopZ2), similarly to nonpathogenic *X. arboricola* strains CFBP 7651, CFBP 7652, CITA 14. CPBF 367, and CPBF 426, which only possess XopAZ and XopR, as observed for nonpathogenic CFBP 7653 and CFBP 7635.

## 4. Discussion

The occurrence of distinct *Xanthomonas* populations colonizing the same host plant has been previously documented [57]. In walnut and stone fruit trees, besides the presence of *X. arboricola* strains belonging to pathovar *juglandis* and *pruni*, characteristic of these two host species, respectively, the isolation of distinct yellow-pigmented xanthomonads has been reported, mostly represented by nonpathogenic lineages that do not form a phylogenetically coherent group with the pathogenic strains of *X. arboricola* pathovars [20,58,59]. This raises the need to understand the role played in the pathosystems by these bacteria characterized by distinct genotypes.

In this study, four *Xanthomonas* strains (CPBF 367, CPBF 424^T^, CPBF 426, CPBF 427) isolated from the same walnut tree were sequenced to provide an in-depth characterization of these co-colonizing strains to disclose differential genomic contents putatively related to pathogenicity, virulence, and other specific niche adaptations. ANI and a core-genome phylogenetic analysis disclosed the presence of two different *Xanthomonas* species in one disease walnut tree, with the confirmation that CPBF 427 belongs to *X. arboricola* pv. *juglandis*, whilst the other three strains, CPBF 367, CPBF 424^T^, and CPBF 426, were already assigned to the recently described species *X. euroxanthea* [30]. Moreover, two of the atypical strains described by Essakhi et al. [20] as *X. arboricola,* CFBP 7635 and CFBP 7653, were now confirmed to belong to *X. euroxanthea*. The role of nonpathogenic strains in *Xanthomonas* evolution and its potential for pathogenicity emergence is often neglected due to their unvalued direct agro-economic impact. However, a recent genomics study on nonpathogenic strains reinforces our lack of knowledge regarding the lifestyle of these strains. In fact, a nonpathogenic isolate from citrus (LMG 8993) was revealed to belong to *X. arboricola* species, being placed phylogenetically closest to nonpathogenic *X. arboricola* isolates from walnut (CFBP 7634 and CFBP 7651), than with other nonpathogenic citrus isolates [60]. Currently, the ecological, evolutionary, and pathogenicity implications of this co-colonization are not understood, but it is hardly refutable that this knowledge is needed for the improvement of efficient phytosanitary practices and the design of appropriate management strategies [61].

The genome size of CPBF 427 (5.23 Mbp) is roughly equal to the genome size reported for other sequenced *X. arboricola* pv. *juglandis* genomes, namely *Xaj* 417 [62], NCPPB 1447 [9], J303 [63], DW3F3, [64], and CFBP 2528^T^ and CFBP 7179 [24] or CFSAN033077 and CFSAN033080 [65]. The genomes of *X. euroxanthea* strains CPBF 367, CPBF 424^T^, and CPBF 426 were smaller (ranging from ≈ 4.90 to 4.97 Mbp), presenting values closer to the nonpathogenic or avirulent strains of *X. arboricola*, i.e., with uncertain pathogenicity or belonging to non-*juglandis*, non-*pruni*, and non-*corylina* pathovars, with genome sizes inferior to 5 Mb [66,67,68]. Despite the presence of one plasmid in strains CPBF 367 and CPBF 426, all the virulence-related homologs were chromosomal. The genetic patrimony of the studied strains illustrated by a Venn diagram encompasses genes associated with basic biological aspects of the *Xanthomonas* genus, as phenotypic traits [69]. Among these, it was possible to discern 212 genes shared exclusively by *X. euroxanthea* strains. The analysis of these gene sets specific to *X. euroxanthea* or *X. arboricola* pv. *juglandis* suggests the presence of genes encoding for proteins associated with biochemical functions that may confer selective advantages, such as adaptation to different niches, pathogenicity, or colonization of a new host [69]. Some genetic determinants of virulence were shown to be group-specific or even strain-specific. For example, *X. euroxanthea* strains evidenced an exclusive presence of the *xpsN* gene, which encodes the XpsN protein of the type II secretion system. Proteins of the xps system were shown to be associated with virulence of *Xanthomonas* species such as *X. campestris*, *X. oryzae*, and *X. euvesicatoria* [7]. Furthermore, the *X. euroxanthea* pathogenic strain CPBF 424^T^ harbors a set of genes that encodes proteins associated with type IV pilus (PilY1, PilX, PilW, PilV, FimT), some of which are considered primary structures of the T4P pilin subunits. Indeed, T4P could play an important role in the pathogenesis of various species of *Xanthomonas*, and in some cases it is thought that this system has a role in plant colonization [54,70]. Comparative studies between pathogenic *X. arboricola* pv. *juglandis* and nonpathogenic *X. arboricola* strains have shown a differential repertoire of genes encoding chemotaxis-related proteins and proteins related to type I, II, and IV secretion systems [24]. Furthermore, a differential repertoire of non-fimbrial adhesins involved in different functions related to bacterial attachment to the host surface were found in all strains, whereas homologs of non-fimbrial adhesins *fhaB* probably associated with the bacteria colonization were only identified in pathogenic strains [24]. In the same way, homologs of proteins involved in the biogenesis of type IV pilus were observed, but the absence of PilA, PilX, and/or PilV proteins in the genomes of pathogenic and nonpathogenic strains may point to the absence of bacterial surface filaments in all strains [24]. Moreover, differences can be pinpointed between pathogenic and nonpathogenic *X. euroxanthea* strains, particularly, the five type IV pilus proteins exclusively present in CPBF 424^T^, and the xylosidases (XylB.1 and XylB.2) only present in CPBF 424^T^ and also in CPBF 427. It was also possible to identify homologs encoding for proteins specific to *X. arboricola* pv. *juglandis* strain CPBF 427 that are missing in *X. euroxanthea*, such as the extracellular enzymes xylosidase (Xsa.1), endoglucanase, polygalacturonase, and degenerate pectate lyase.

Furthermore, major genomic differences between the strains analyzed in this study were observed for T3SS and related T3E homologs. In *Xanthomonas* spp., T3SS is crucial for translocating effector proteins that have a key role in bacterial proliferation in host tissues and the development of disease symptoms [21]. The majority of pathogenic strains from the 44 analyzed genomes, including CPBF 427 and *X. euroxanthea* CPBF 424^T^, displayed a T3SS profile comprised of most homologous genes for highly conserved T3SS of the Hrp2 family [23,24,25,58,71]. Interestingly, a similar pattern was spotted for nonpathogenic *X. arboricola* strains CFBP 7652, CFBP 7651, and CITA 14 and for the pathogenic *X. euroxanthea* strain CPBF 424^T^, with the exception for T3SS homologs HrpF and HrpW, which were slightly below the 75% query coverage threshold used to filter for the most conserved homologs. Conversely, nonpathogenic *X. euroxanthea* CPBF 367 and CPBF 426, and nonpathogenic *X. arboricola* CFBP 7653, CFBP 7645, CFBP 7635, CFBP 7634, CFBP 7629, CITA 44, and CITA 124 lacked most of the genes coding for the macromolecular structure of T3SS, as well as the *hrpF* gene, involved in the translocation of T3E [24], despite harboring regulators genes of T3SS, as *hrpX* and *hrpG* [72,73]. Interestingly CPBF 424^T^ lacks homologs for several pathogenicity genes thought to be essential for *X. arboricola* pv. *juglandis* strains. In fact, CPBF 424^T^ harbors homologs for seven known effectors, i.e., less than the nine to ten T3Es found in nonpathogenic *X. arboricola* CFBP 7651, CFBP 7652, and CITA 14. Only two T3Es were identified in nonpathogenic *X. euroxanthea* stains (CPBF 367 and CPBF 426), suggesting that *X. euroxanthea* may also make use of other virulence and pathogenicity-related proteins to trigger infection. Still, the intricate mechanism for successful pathogenicity of *X. euroxanthea* CPBF 424^T^ can only be disclosed with further investigation and dedicated functional assays.

Cesbron et al. [24] and Garita-Cambronero et al. [71] started to elucidate the mechanisms associated with the emergence of *X. arboricola* pv. *juglandis* and *X. arboricola* pv. *pruni* pathogenic strains. This was achieved by comparative genomics of *X. arboricola* strains differing on their pathogenicity, including the nonpathogenic *X. arboricola* strains isolated from walnut and evaluated in this study (CFBP 7634 and CFBP 7651), and *X. arboricola* strains (CFBP 14, CFBP 44, and CFBP 124) isolated from *Prunus*. Regardless of the contributions of these comparative genomics studies in highlighting the importance of T3SS and T3E genes, dedicated functional studies are still required to identify essential genes for successful infection. Furthermore, these nonpathogenic strains are particularly valuable to address questions regarding pathogenicity evolution in *Xanthomonas*.

## 5. Conclusions

The extensive genomic comparison of four walnut-associated strains isolated from the same walnut specimen and belonging to two species (*X. arboricola* pv. *juglandis* and *X. euroxanthea*), including pathogenic (CPBF 427 and CPBF 424^T^) and nonpathogenic strains (CPBF 367 and CPBF 426), provides insights about niche-specific adaptations that could inform on the role played by each of these strains in the co-colonization of walnut. Comprehensive genomics analysis, which also includes previously reported nonpathogenic *X. arboricola* strains, shows that two of these strains, CFBP 7635 and CFBP 7653, belong to *X. euroxanthea* species. The data gathered suggest a pattern of homologous genes putatively associated with pathogenicity, virulence, and niche-specific adaptations that need to be addressed in future functional studies to determine their importance in walnut diseases caused by *Xanthomonas*.

## Figures and Tables

**Table 1:** General genomic features of the four strains analyzed in this study.

General Features	*Xanthomonas euroxanthea* Strains			*Xanthomonas arboricola* pv. *juglandis* Strain
	CPBF 367	CPBF 424T	CPBF 426	CPBF 427
Genome size (bp)	4,968,459	4,900,930	4,900,648	5,228,174
Contigs	2	1	2	1
N50 (bp)	4,923,218	4,900,930	4,883,254	5,228,174
G+C content (%)	65.81	65.88	65.85	65.38
Plasmids	1	0	1	0
Total genes	4157	4119	4143	4465
Total CDSs	4077	4040	4066	4367
Coding genes	4012	3993	4003	4237
RNA genes	80	79	77	98
rRNA (5S, 16S, 23S)	2, 2, 2	2, 2, 2	2, 2, 2	2, 2, 2
ncRNA	18	17	18	38
tRNA	56	56	53	54
Pseudogenes	65	47	63	130
ENA/GenBank accession number	GCA_903989455	CGA_905187425	GCA_903989465	GCA_903989475
Reference	[32]	This study	[32]	[34]

**Table 2:** Comparison of the occurrence of homologs associated with chemotaxis, non-fimbrial adhesins, T2SS, extracellular enzymes, and T4P, between *Xanthomonas euroxanthea* strains (CPBF 367, CPBF 424^T^, and CPBF 426) and the *Xanthomonas arboricola* pv. *juglandis* (*Xaj*) strain (CPBF 427).

			*X. euroxanthea*			*Xaj*
Protein/Gene Name	Label	GenBank Accession Number	CPBF 367	CPBF 424^T^	CPBF 426	CPBF 427
**Chemotaxis-related proteins:**						
Methyl-accepting chemotaxis protein	XCV1938	CAJ23615.1	● *	●	●	○
Chemotaxis protein	XAC3768	AAM38611.1	●	●	●	○
**Non-fimbrial adhesins:**						
Filamentous hemagglutinin-related protein (*fhaB1*)	FhaB1	CAJ23537.1	●	○	○	●
Filamentous hemagglutinin-related protein (*fhaB2*)	FhaB2	CAJ23538.1	●	○	○	●
**Type II secretion system (T2SS):**						
General secretion pathway protein XpsN (*xpsN*)	XpsN	WP_011035909.1	●	●	●	○
**Extracellular enzymes:**						
Xylosidase/arabinosidase (*xylB*)	XylB.1	WP_011036375.1	○	●	○	●
Xylosidase/arabinosidase (*xsa*)	Xsa.1	WP_011037540.1	○	○	○	●
Xylosidase/arabinosidase (*xylB*)	XylB.2	WP_011039174.1	○	●	○	●
Endoglucanase (*bcsZ*)	bcsZ	AAM38359.1	○	○	○	●
Polygalacturonase (*pglA*)	pglA	WP_011037410.1	○	○	○	●
Pectate lyase E (*pelA*)	pelA	WP_011035380.1	●	●	●	○
Degenerated pectate lyase (*pel*)	pel	AAM37225.1	○	○	○	●
Pectinesterase	XCC0121	WP_011035379.1	●	●	●	○
**Type IV pilus (T4P):**						
PilY1 protein (*pilY1*)	pilY1	WP_011051753.1	○	●	○	○
PilX protein (*pilX*)	pilX	WP_011051754.1	○	●	○	○
PilW protein (*pilW*)	XAC2667	WP_040107776.1	○	●	○	○
Pre-pilin leader sequence (*pilV*)	pilV	WP_011051756.1	○	●	○	○
Pre-pilin like leader sequence (*fimT*)	fimT	WP_011051757.1	○	●	○	○

* ●, present; ○, not present.

## Data Availability

The assembled genome sequences have been deposited in the European Nucleotide Archive (ENA) under the accession numbers GCA_903989455, CGA_905187425, GCA_903989465, and GCA_903989475 for CPBF 367, CPBF 424^T^, CPBF 426, and CPBF 427, respectively.

## Supplementary Materials

The following are available online at https://www.mdpi.com/2076-2607/9/3/624/s1, Figure S1. (a) Scheme representing presence/absence of type 2 secretion system (T2SS) putative homologs in 44 Xanthomonas spp. genomes; (b) BLAST identity (%) and length (i.e., % of query coverage) for type 2 secretion system (T2SS) putative homologs in CPBF 367, CPBF 424T, CPBF 426, and CPBF 427, Figure S2. (a) Scheme representing presence/absence of type 4 pilus (T4P) putative homologs in 44 Xanthomonas spp. genomes; (b) BLAST identity (%) and length (i.e., % of query coverage for type 4 pilus T4P) putative homologs in CPBF 367, CPBF 424T, CPBF 426, and CPBF 427, Figure S3. (a) Scheme representing presence/absence of non-fimbrial adhesin putative homologs in 44 Xanthomonas spp. genomes; (b) BLAST identity (%) and length (i.e., % of query coverage) of non-fimbrial adhesins putative homologs in CPBF 367, CPBF 424T, CPBF 426, and CPBF 427, Figure S4. (a) Scheme representing presence/absence of chemotaxis-related protein putative homologs in 44 Xanthomonas spp. genomes; (b) BLAST identity (%) and length (i.e., % of query coverage) of chemotaxis-related protein putative homologs in CPBF 367, CPBF 424T, CPBF 426, and CPBF 427, Figure S5. (a) Scheme representing presence/absence of extracellular enzyme putative homologs in 44 Xanthomonas spp. genomes; (b) BLAST identity (%) and length (i.e., % of query coverage) of extracellular enzyme putative homologs in CPBF 367, CPBF 424T, CPBF 426, and CPBF 427, Figure S6. (a) BLAST identity (%) and length (i.e., % of query coverage) of type 3 secretion system (T3SS) putative homologs in CPBF 367, CPBF 424T, CPBF 426, and CPBF 427; Figure S6. (b) BLAST identity (%) and length (i.e., % of query coverage) of type 3 effector (T3E) putative homologs in CPBF 367, CPBF 424T, CPBF 426. and CPBF 427. Table S1: List of Xanthomonas spp. genomes used in this study, Table S2: Average nucleotide identity results for the genomes of the 44 strains analyzed in this study, Table S3: Best BLAST hit results of putative homologs.
