# Supplementary material for: Comparative Genomics of Xanthomonas euroxanthea and Xanthomonas arboricola pv. juglandis Strains Isolated from a Single Walnut Host Tree
Source: Microorganisms. 2021 Mar 17;9(3):624. doi: 10.3390/microorganisms9030624 (PMC8003016; doi:10.3390/microorganisms9030624)
Supplement: Supplementary file 1 [file microorganisms-09-00624-s001.zip › Supplementary material_proof/Supplementar material_proof.pdf]

microorganisms

MDPI

Article supplementary materials

Comparative genomics of *Xanthomonas euroxanthea* and *Xanthomonas arboricola* pv. *juglandis* strains isolated from a single walnut host tree

Camila Fernandes, Leonor Martins, Miguel Teixeira, Jochen Blom, Joël F. Pothier, Nuno A. Fonseca and Fernando Tavares

Supplemental tables:

Table S1: List of *Xanthomonas* spp. genomes used in this study.

Table S2: Average Nucleotide Identity results for the genomes of the 44 strains analyzed in this study (not included in this file).

Table S3: Best BLAST hit results putative homologs (not included in this file).

Supplemental figures:

Figure S1(a). Scheme representing presence absence for type 2 secretion system (T2SS) putative homologs.

Figure S1(b). BLAST identity (%) and length (i.e. % of query coverage) for type 2 secretion system (T2SS) putative homologs in CPBF 367, CPBF 424T, CPBF 426 and CPBF 427.

Figure S2(a). Scheme representing presence absence for type 4 pilus (T4P) putative homologs.

Figure S2(b). BLAST identity (%) and length (i.e. % of query coverage for type 4 pilus T4P) putative homologs in CPBF 367, CPBF 424^{T}, CPBF 426 and CPBF 427.

Figure S3(a). Scheme representing presence absence Non fimbrial adhesins putative homologs.

Figure S3(b). BLAST identity (%) and length (i.e. % of query coverage) for Non fimbrial adhesins putative homologs in CPBF 367, CPBF 424^{T}, CPBF 426 and CPBF 427.

Figure S4(a). Scheme representing presence absence for Chemotaxis related proteins putative homologs.

Figure S4(b). BLAST identity (%) and length (i.e. % of query coverage) for Chemotaxis related proteins putative homologs in CPBF 367, CPBF 424^{T}, CPBF 426 and CPBF 427.

Figure S5(a). Scheme representing presence absence for Extracellular enzymes putative homologs.

Figure S5(b). BLAST identity (%) and length (i.e. % of query coverage) for Extracellular enzymes putative homologs in CPBF 367, CPBF 424^{T}, CPBF 426 and CPBF 427.

Figure S6(a). BLAST identity (%) and length (i.e. % of query coverage) for type 3 secretion system(T3SS) putative homologs in CPBF 367, CPBF 424T, CPBF 426 and CPBF 427.

Figure S6(b). BLAST identity (%) and length (i.e. % of query coverage) for type 3 effectors (T3E) putative homologs in CPBF 367, CPBF 424^{T}, CPBF 426 and CPBF 427.

Table S1. List of *Xanthomonas* spp. genomes used in this study

Strain	Acession number	Assembly accession	Label
Xanthomonas arboricola 3004	NZ_AZQY01000132	GCF_000585435.1	Xa3004
Xanthomonas arboricola CFBP 1022	NZ_MDRU01000000	GCF_002940165.1	Xa1022
Xanthomonas arboricola CFBP 7629	NZ_MIGI00000000.1	GCF_002940425.1	Xa7629
Xanthomonas arboricola CFBP 7634	NZ_JZEH01000004	GCF_001013485.1	Xa7634
Xanthomonas arboricola CFBP 7635	NZ_JACHNI000000000.1	GCF_014198915.1	Xa7635
Xanthomonas arboricola CFBP 7645	NZ_MIGY00000000.1	GCF_002940665.1	Xa7645
Xanthomonas arboricola CFBP 7651	NZ_JZEI01000006	GCF_001013505.1	Xa7651
Xanthomonas arboricola CFBP 7652	NZ_MIGJ00000000.1	GCF_002940445.1	Xa7652
Xanthomonas arboricola CFBP 7653	MIGK00000000.1	GCA_002940465.1	Xa7653
Xanthomonas arboricola CITA 124	NZ_LXKK01000128	GCF_001674995.1	Xa-CITA124
Xanthomonas arboricola CITA 14	NZ_LXIB01000072	GCF_001675005.1	Xa-CITA14
Xanthomonas arboricola CITA 44	NZ_LJGM01000071	GCF_001306965.1	Xa-CITA44
Xanthomonas arboricola pv. *arracaciae* CFBP 7407^{PT}	NZ_MIGU01000232	GCF_002940565.1	Xa-a7407
Xanthomonas arboricola pv. *celebensis* NCPPB 1630	NZ_KL638873	GCF_000724915.1	Xa-c1630
Xanthomonas arboricola pv. *celebensis* NCPPB 1832^{PT}	NZ_KL638866	GCF_000724925.1	Xa-c1832
Xanthomonas arboricola pv. *corylina* NCCB 100457	NZ_APMC02000002	GCF_000355635.2	Xa100457
Xanthomonas arboricola pv. *fragariae* CFBP 6773	NZ_OEQD01000021	GCF_900240345.1	Xaf-CFBP6773
Xanthomonas arboricola pv. *fragariae* LMG 19145^{PT}	NZ_OEQL01000022	GCF_900240435.1	Xaf-LMG19145
Xanthomonas arboricola pv. *juglandis* 417*	NZ_CP012251	GCF_001237985.1	Xaj417*
Xanthomonas arboricola pv. *juglandis* CFBP 2528^{T}	NZ_JZEF00000000	GCF_001013475.1	Xa2528
Xanthomonas arboricola pv. *juglandis* CFBP 7179	NZ_JZEG01000014	GCF_001013495.1	Xa7179
Xanthomonas arboricola pv. *juglandis* DW3F3	NZ_PNRC01000008	GCF_002879695.1	XaDW3F3
Xanthomonas arboricola pv. *juglandis* J303	NZ_LSGZ01000161	GCF_001643295.1	XaJ303
Xanthomonas arboricola pv. *juglandis* NCPPB 1447	NZ_AJTL01000371	GCF_000306055.1	Xaj1447
Xanthomonas arboricola pv. *juglandis* CPBF 427*	SAMEA7068332	GCA_903989475	Xaj427*
Xanthomonas arboricola pv. *populi* CFBP 3122	NZ_MIGV01000092	GCF_002940585.1	Xa3122
Xanthomonas arboricola pv. *populi* CFBP 3123^{PT}	NZ_MDEB00000000.1	GCF_002939945.1	Xa3123
Xanthomonas arboricola pv. *pruni* CFBP 3894^{PT}	NZ_LOMI01000077	GCF_001741965.1	Xa3894
Xanthomonas arboricola pv. *pruni* IVIA 2626 1	NZ_LJGN01000099	GCF_001306955.1	Xa2626
Xanthomonas arboricola pv. *zantedeschiae* CFBP 7410^{PT}	NZ_MIGW01000037	GCF_002940625.1	Xa-z7410
Xanthomonas axonopodis Xac29-1*	NC_020800	GCF_000348585.1	Xax29-1*
Xanthomonas citri subsp. *citri* A306*	NZ_CP006857	GCF_000816885.1	XciA306*
Xanthomonas fuscans subsp. *fuscans* 4834-R*	NZ_FO681494	GCF_000969685.2	Xfu4834-R*
Xanthomonas euroxanthea CPBF 367*	SAMEA7068330	GCA_903989455	Xe367*
Xanthomonas euroxanthea CPBF 424^{T}*	SAMEA7742038	GCA_905187425	Xe424*
Xanthomonas euroxanthea CPBF 426*	SAMEA7068331	GCA_903989465	Xe426*
Xanthomonas euvesicatoria LMG 930*	NZ_CP018467	GCF_001908795.1	XeuLMG930*
Xanthomonas fragariae fap21*	NZ_CP016830	GCF_001705565.1	Xfap21*
Xanthomonas gardneri ICMP 7383*	NZ_CP018731	GCF_001908775.1	Xg7383*
Xanthomonas hortorum pv. *carotae* M081*	NZ_CM002307	GCF_000505565.1	XhcM081*
Xanthomonas oryzae pv. *oryzicola* CFBP 2286 *	NZ_CP011962	GCF_001042735.1	Xo2286*
Xanthomonas prunicola CFBP 8353^{T}	NZ_PHKV00000000	GCF_002846205.1	Xp8353
Xanthomonas prunicola CFBP 8354	NZ_PHKW00000000	GCF_002846225.1	Xp8354
Xanthomonas prunicola CFBP 8355	NZ_PHKX00000000	GCF_002846195.1	Xp8355

* Chromossomes in a single contig

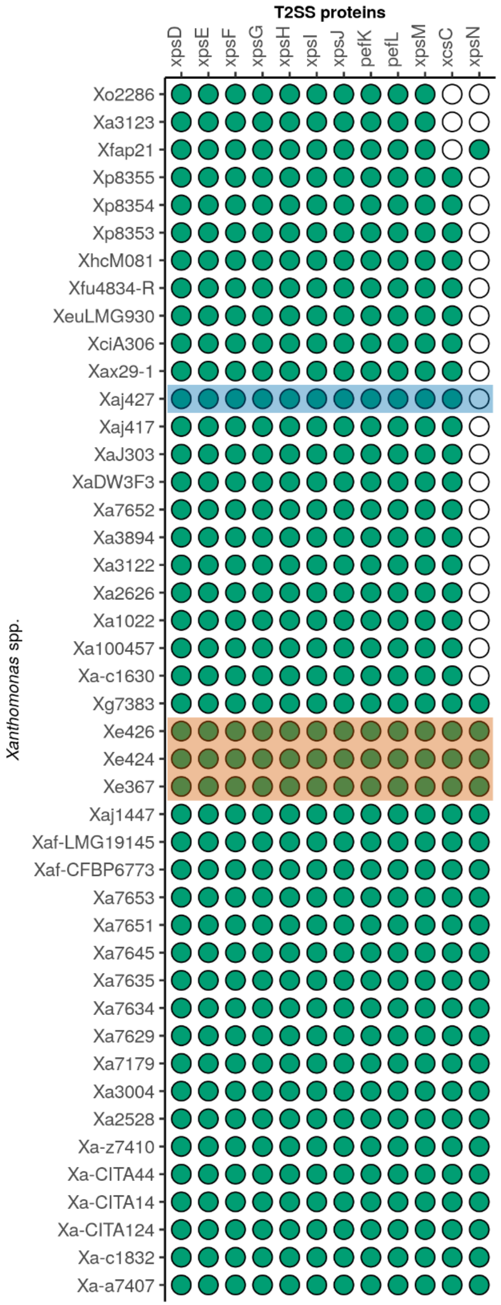

Figure S1(a). Scheme representing presence/absence for type 2 secretion system (T2SS) putative homologs in 44 *Xanthomonas* spp. genomes; ●, present; ○, not present; considering a tBLASTn hit with a query length similarity threshold ≥75%, and sequence identity with ≥ 40% cut-off. Results for *X. euroxanthea* strains CPBF 367, CPBF 424^{T} and CPBF 426 are highlighted in orange and for *X. arboricola* pv. *juglandis* CPBF 427 in blue. The strain names refer to the code field from Table S1. Best blast results and accession numbers of sequences used as query are disclosed in Table S3.

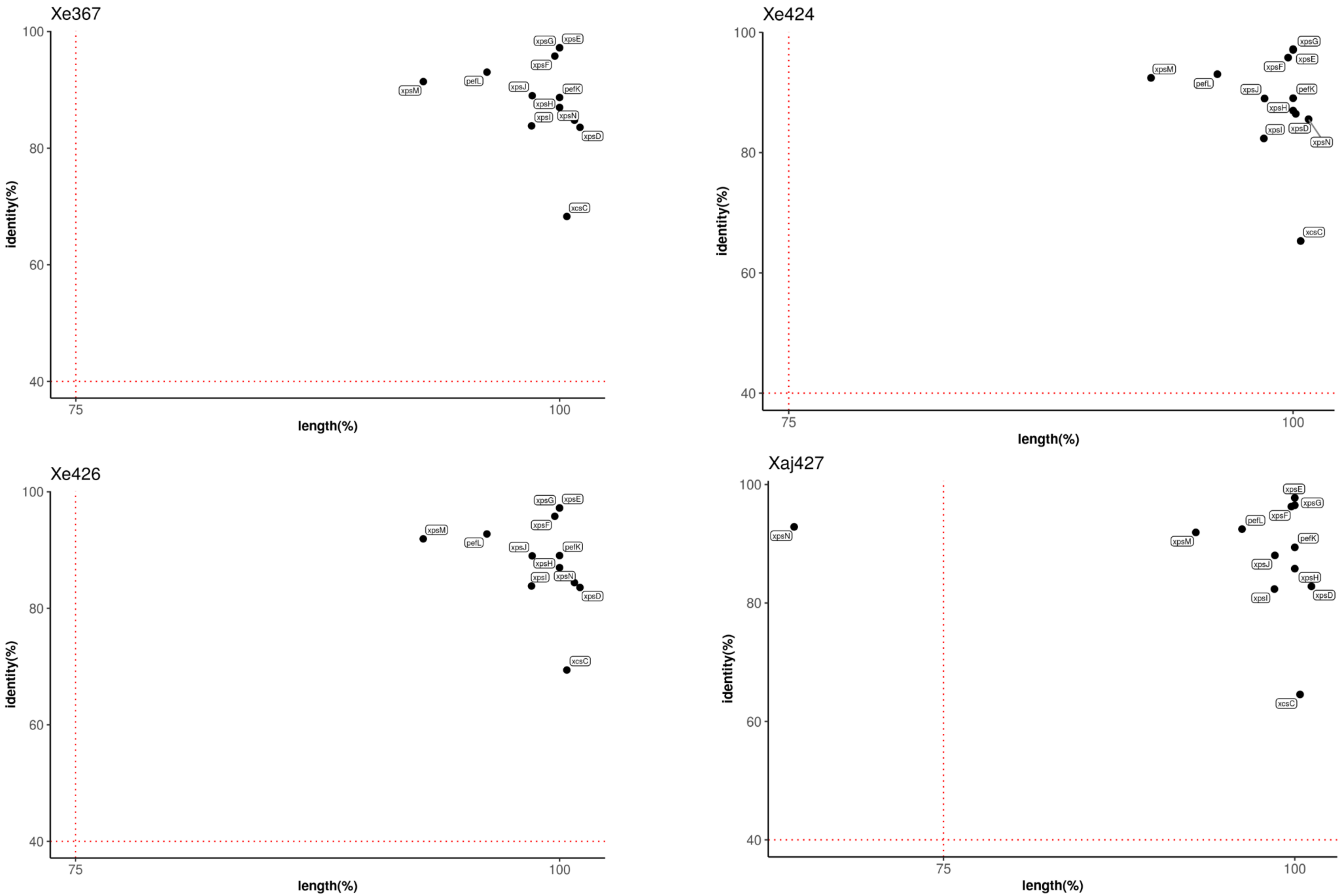

Figure S1(b). BLAST identity (%) and length (i.e. % of query coverage) for type 2 secretion system (T2SS) putative homologs in CPBF 367, CPBF 424^{T}, CPBF 426 and CPBF 427. Red lines delineate the applied threshold of 40% identity and 75% query coverage. Best blast results and accession numbers of sequences used as query are disclosed in Table S3.

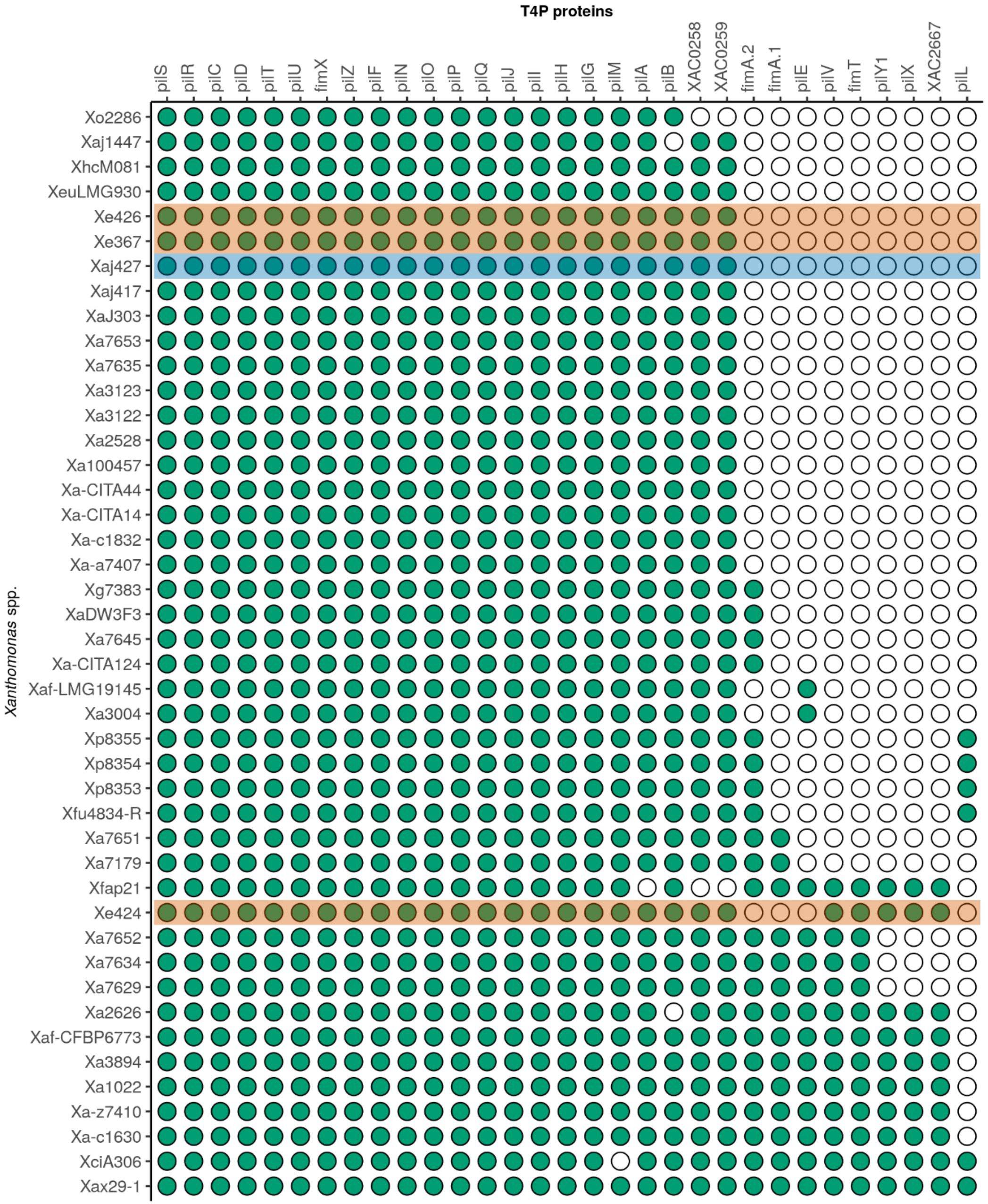

Figure S2(a). Scheme representing presence/absence for type 4 pilus (T4P) putative homologs in 44 *Xanthomonas* spp. genomes; ●, present; ○, not present; considering a tBLASTn hit with a query length similarity threshold ≥75%, and sequence identity with ≥ 40% cut-off. Results for *X. euroxanthea* strains CPBF 367, CPBF 424^T and CPBF 426 are highlighted in orange and for *X. arboricola* pv. *juglandis* CPBF 427 in blue. The strain names refer to the code field from Table S1. Best blast results and accession numbers of sequences used as query are disclosed in Table S3.

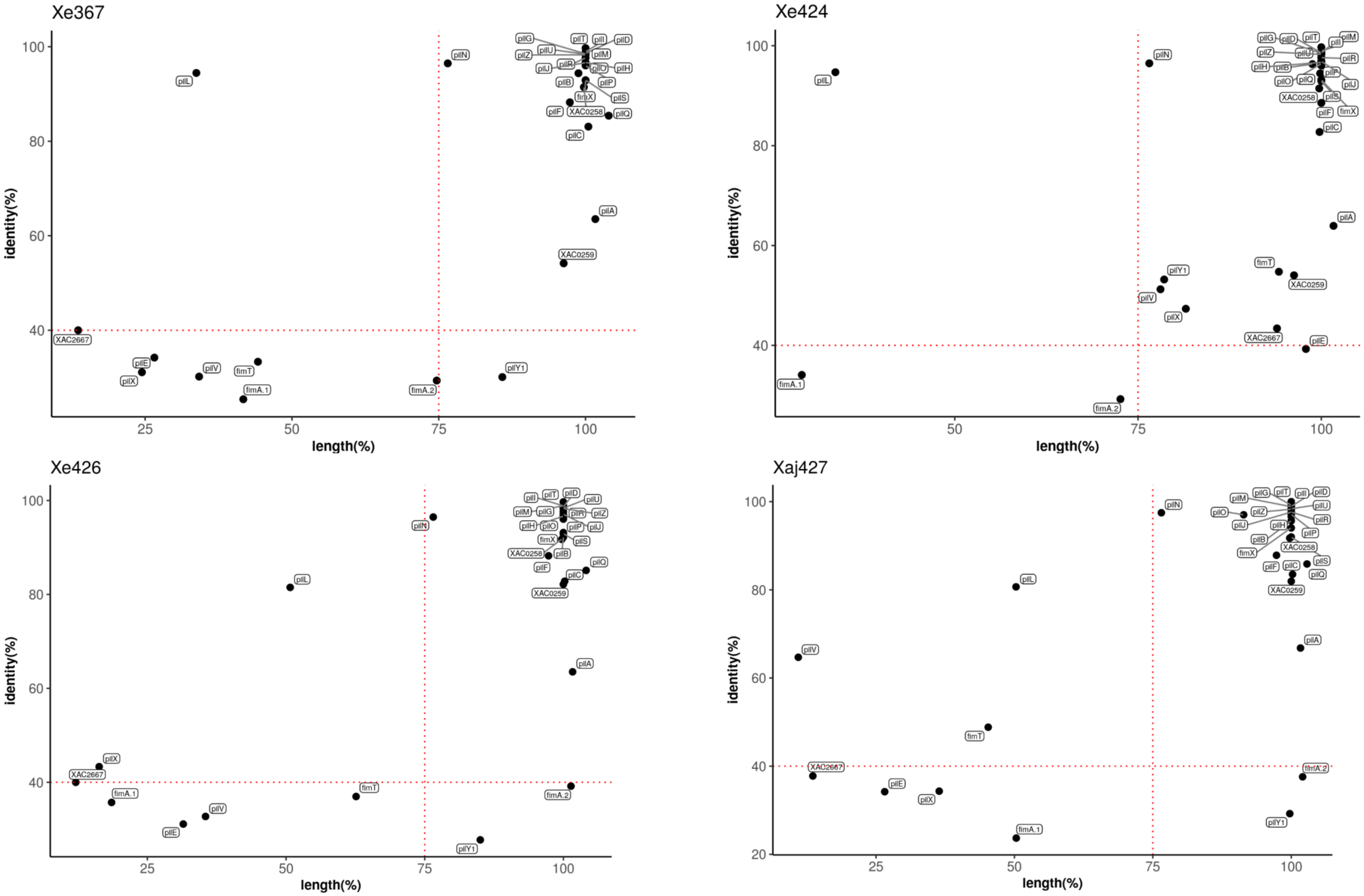

Figure S2(b). BLAST identity (%) and length (i.e. % of query coverage) for type 4 pilus (T4P) putative homologs in CPBF 367, CPBF 424^{T}, CPBF 426 and CPBF 427. Red lines delineate the applied threshold of 40% identity and 75% query coverage. Best blast results and accession numbers of sequences used as query are disclosed in Table S3.

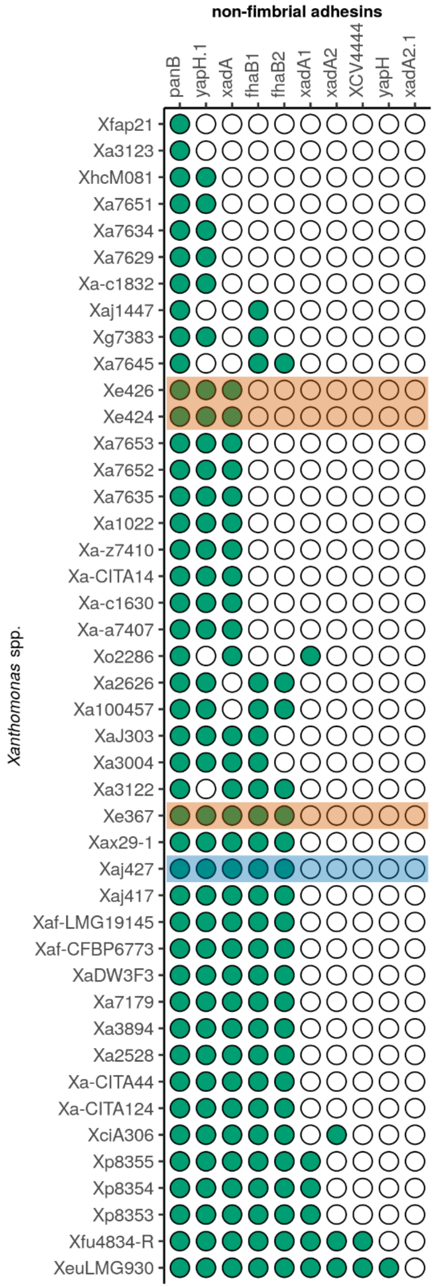

Figure S3(a). Scheme representing presence/absence Non-fimbrial adhesins putative homologs in 44 *Xanthomonas* spp. genomes; ●, present; ○, not present; considering a tBLASTn hit with a query length similarity threshold ≥75%, and sequence identity with ≥ 40% cut-off. Results for *X. euroxanthea* strains CPBF 367, CPBF 424^T and CPBF 426 are highlighted in orange and for *X. arboricola* pv. *juglandis* CPBF 427 in blue. The strain names refer to the code field from Table S1. Best blast results and accession numbers of sequences used as query are disclosed in Table S3.

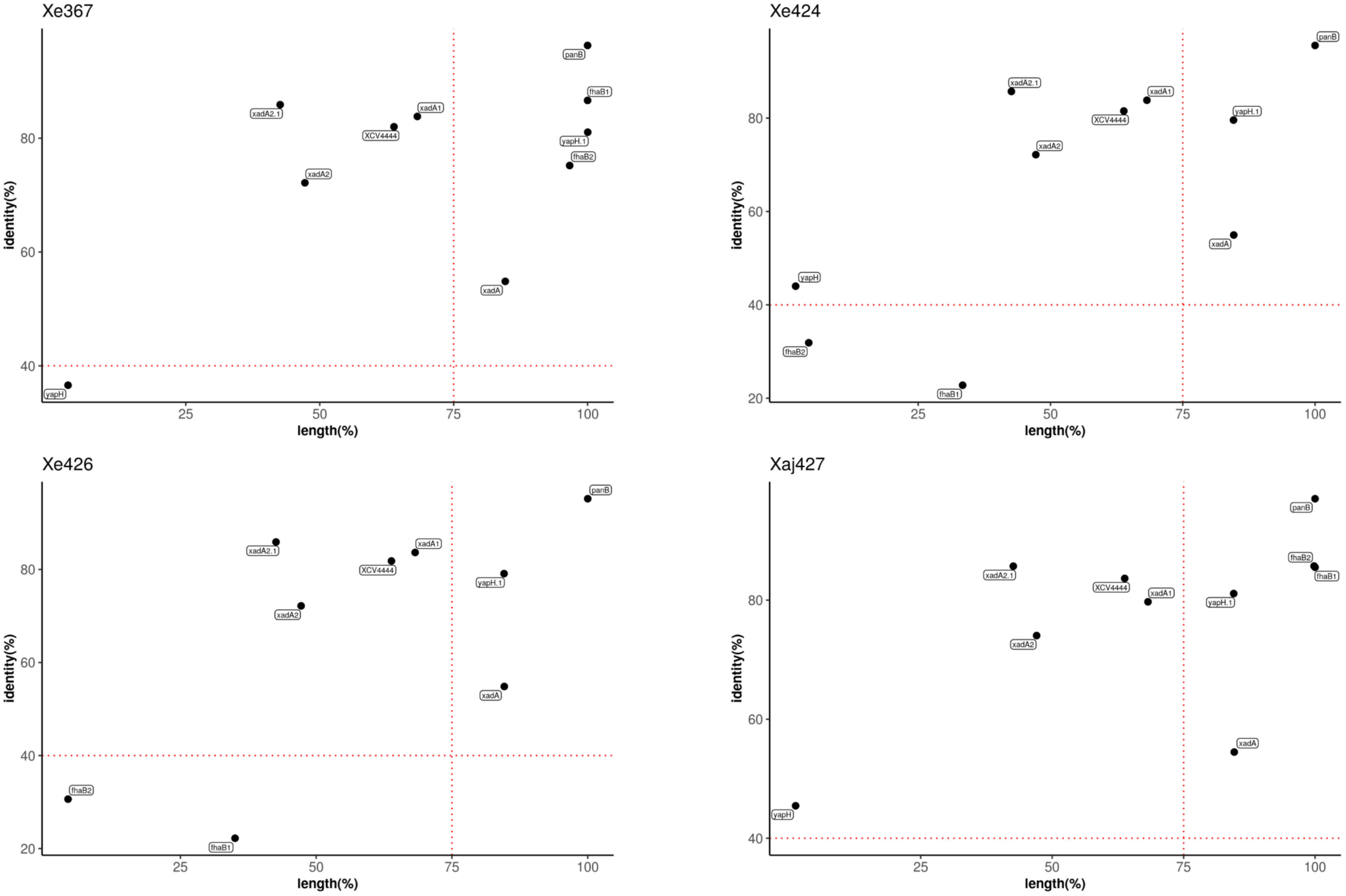

Figure S3(b). BLAST identity (%) and length (i.e. % of query coverage) for Non-fimbrial adhesins putative homologs in CPBF 367, CPBF 424^{T}, CPBF 426 and CPBF 427. Red lines delineate the applied threshold of 40% identity and 75% query coverage. Best blast results and accession numbers of sequences used as query are disclosed in Table S3.

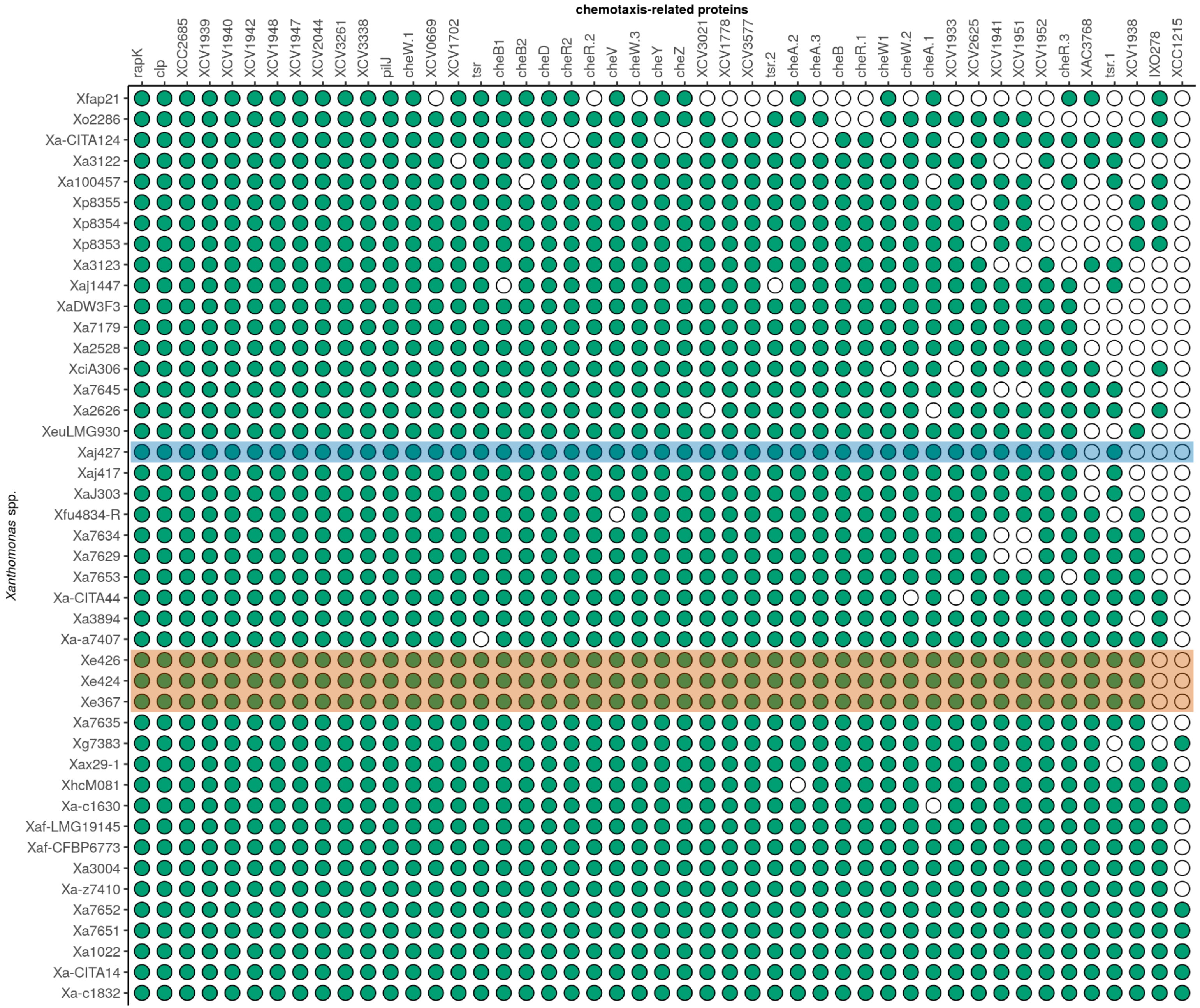

Figure S4(a). Scheme representing presence/absence for Chemotaxis-related proteins putative homologs in 44 *Xanthomonas* spp. genomes; ●, present; ○, not present; considering a tBLASTn hit with a query coverage threshold ≥75%, and sequence identity with ≥ 40% cut-off. Results for *X. euroxanthea* strains CPBF 367, CPBF 424^{T} and CPBF 426 are highlighted in orange and for *X. arboricola* pv. *juglandis* CPBF 427 in blue. The strain names refer to the code field from Table S1. Best blast results and accession numbers of sequences used as query are disclosed in Table S3.

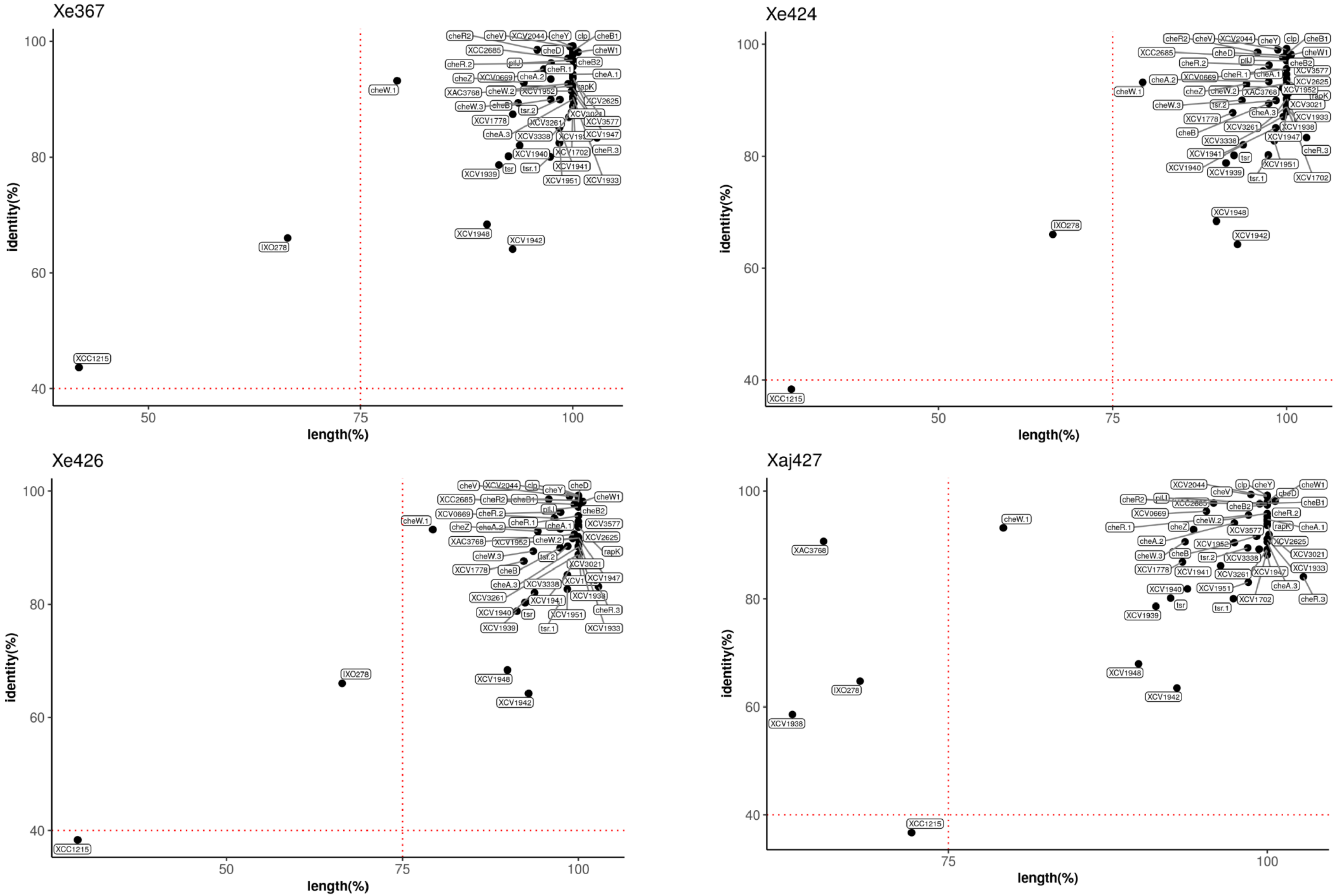

Figure S4(b). BLAST identity (%) and length (i.e. % of query coverage) for Chemotaxis-related proteins putative homologs in CPBF 367, CPBF 424[T], CPBF 426 and CPBF 427. Red lines delineate the applied threshold of 40% identity and 75% query coverage. Best blast results and accession numbers of sequences used as query are disclosed in Table S3.

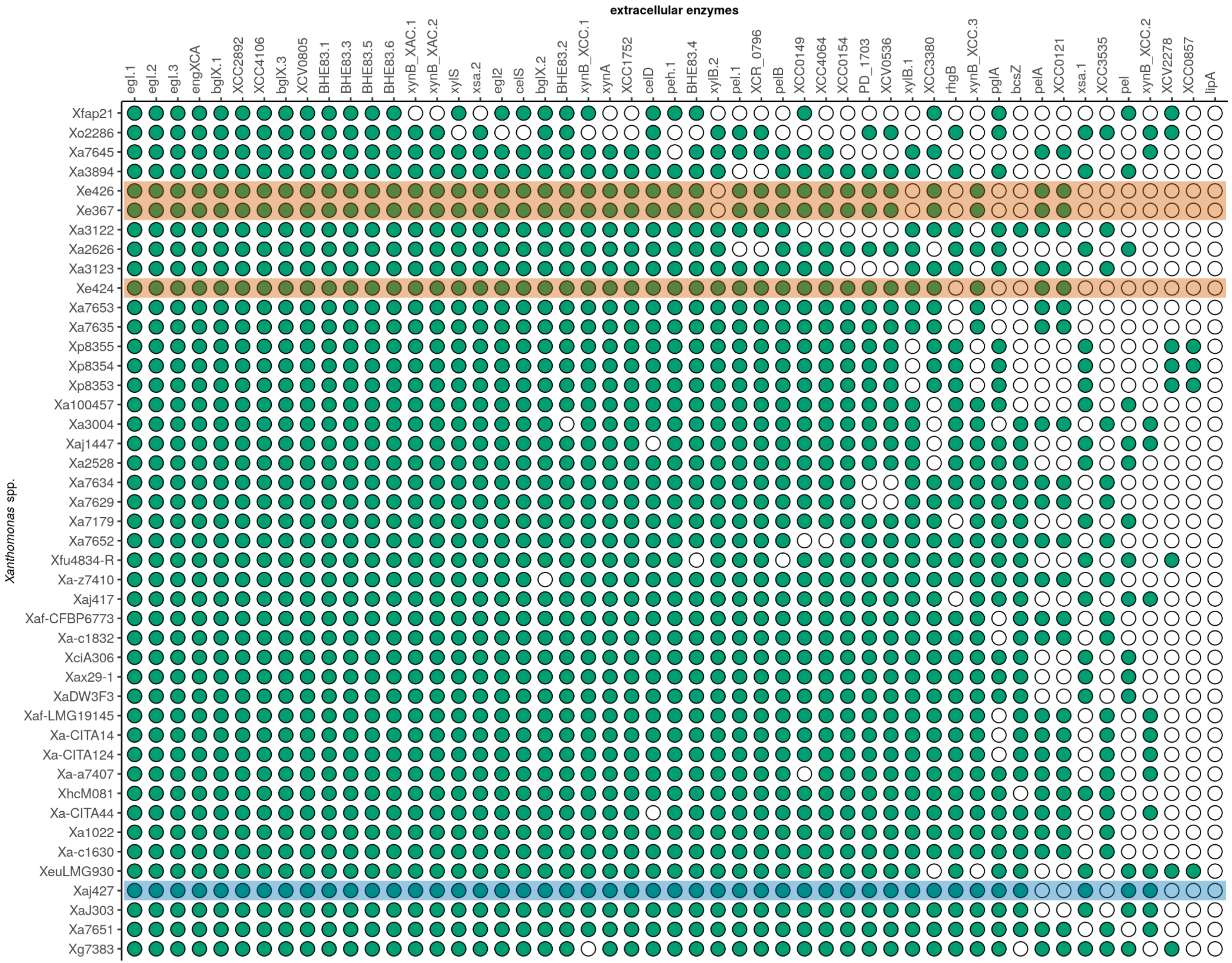

Figure S5(a). Scheme representing presence/absence for Extracellular enzymes putative homologs in 44 *Xanthomonas* spp. genomes; ●, present; ○, not present; considering a tBLASTn hit with a query length similarity threshold ≥75%, and sequence identity with ≥ 40% cut-off. Results for *X. euroxanthea* strains CPBF 367, CPBF 424^{T} and CPBF 426 are highlighted in orange and for *X. arboricola* pv. *juglandis* CPBF 427 in blue. The strain names refer to the code field from Table S1. Best blast results and accession numbers of sequences used as query are disclosed in Table S3.

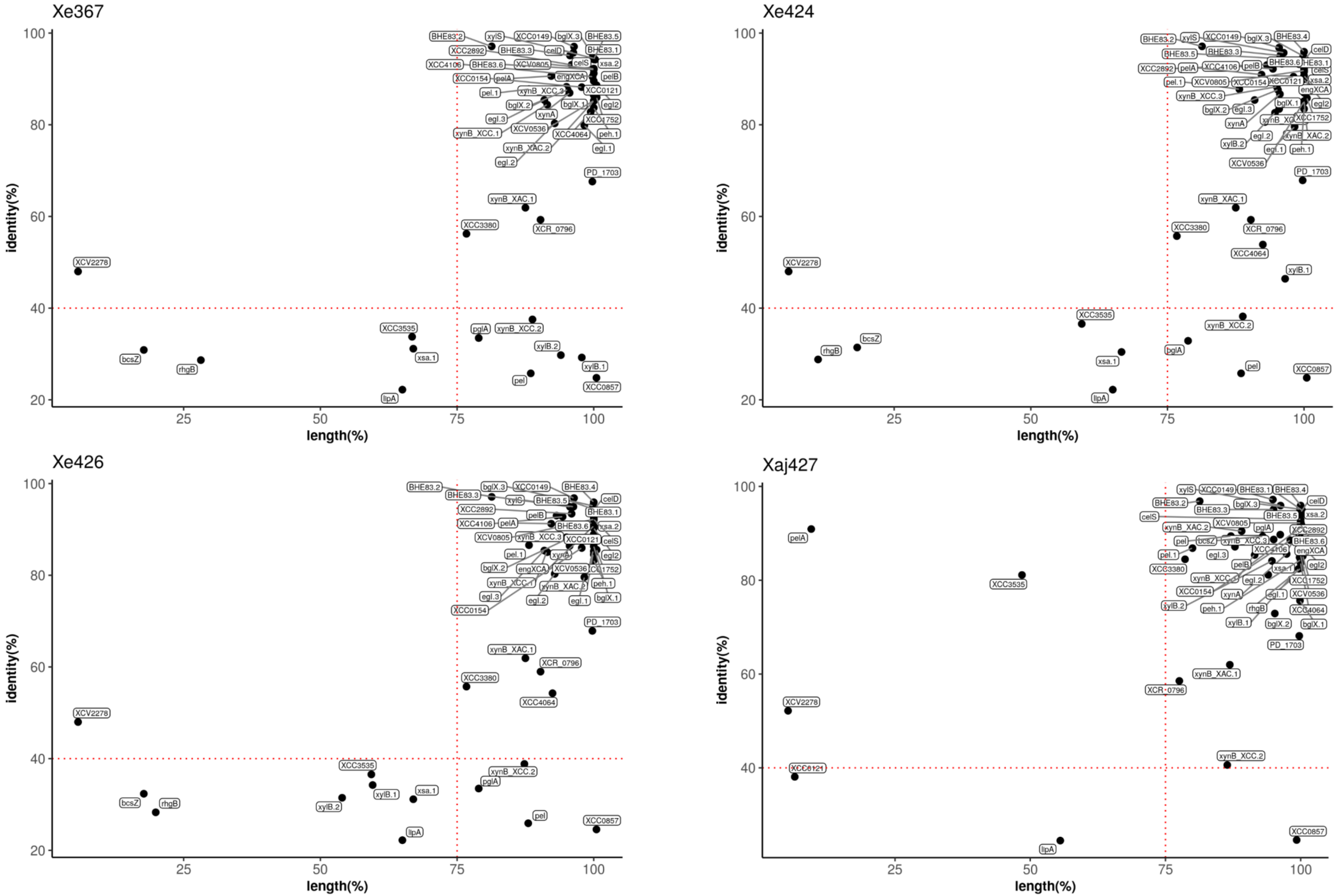

Figure S5(b). BLAST identity (%) and length (i.e. % of query coverage) for Extracellular enzymes putative homologs in CPBF 367, CPBF 424^{T}, CPBF 426 and CPBF 427. Red lines delineate the applied threshold of 40% identity and 75% query coverage. Best blast results and accession numbers of sequences used as query are disclosed in Table S3.

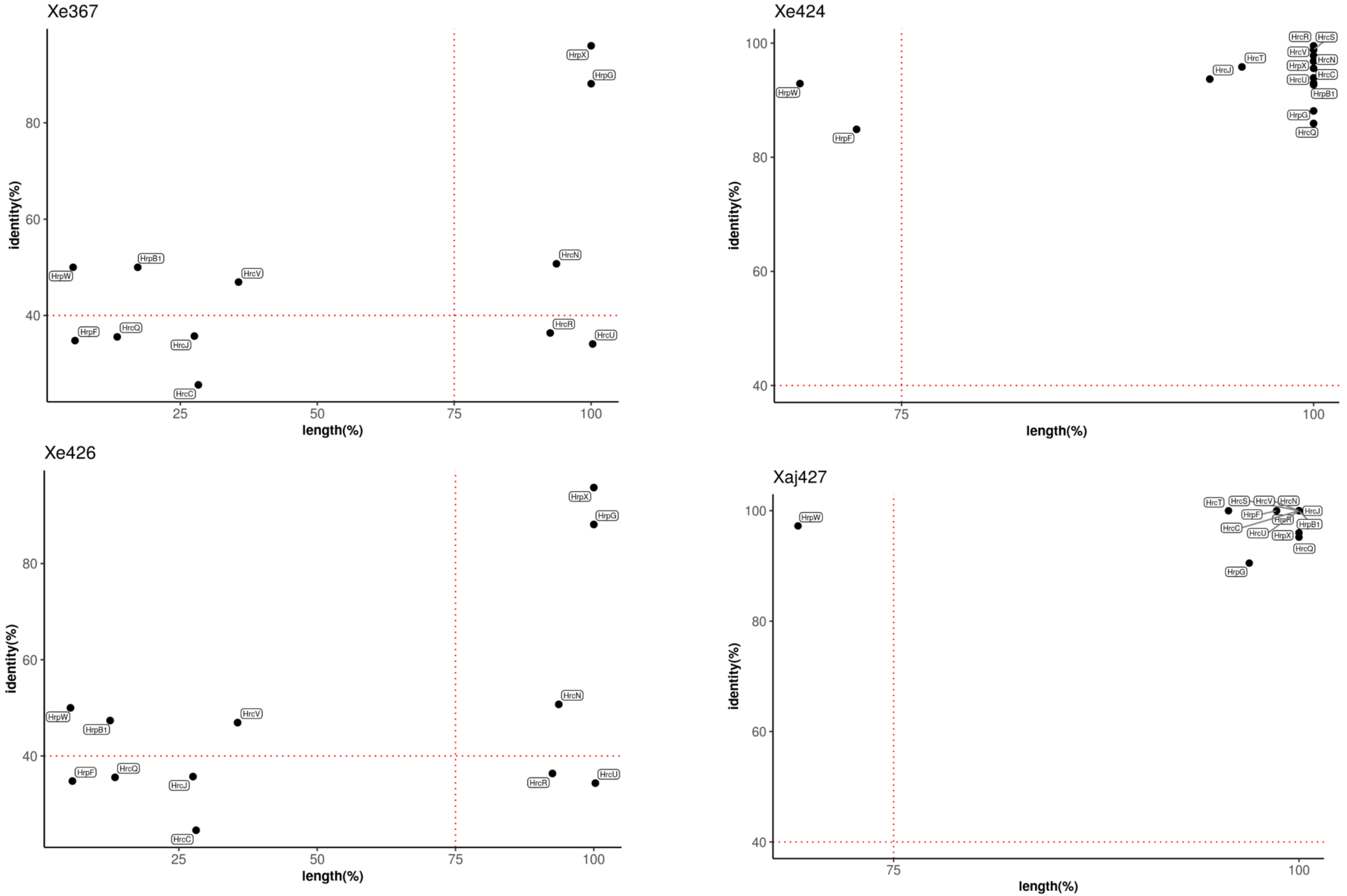

Figure S6(a). BLAST identity (%) and length (i.e. % of query coverage) for type 3 secretion system (T3SS) putative homologs in CPBF 367, CPBF 424^{T}, CPBF 426 and CPBF 427. Red lines delineate the applied threshold of 40% identity and 75% query coverage. Best blast results and accession numbers of sequences used as query are disclosed in Table S3.

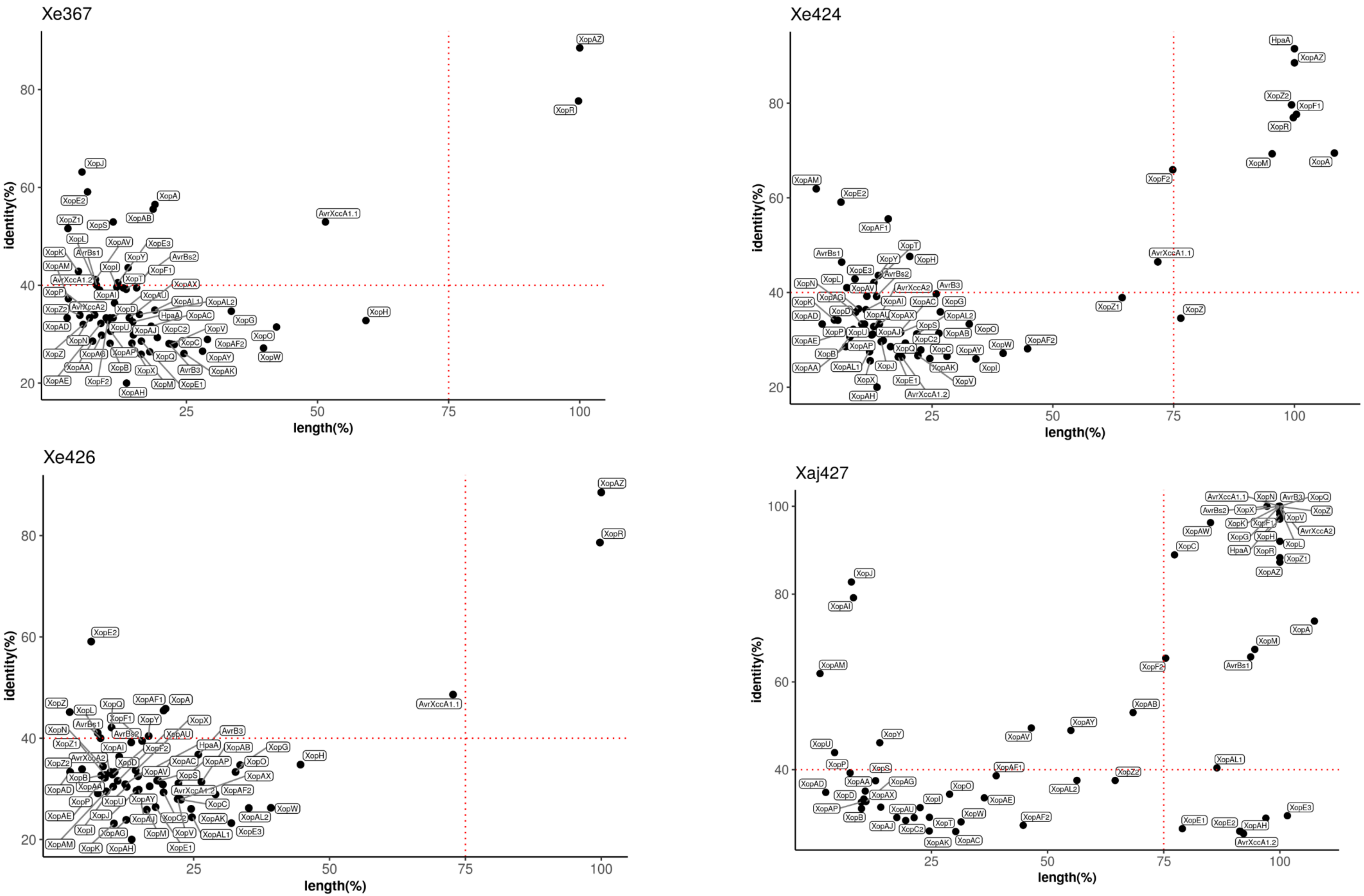

Figure S6(b). BLAST identity (%) and length (i.e. % of query coverage) for type 3 effectors (T3E) putative homologs in CPBF 367, CPBF 424^{T}, CPBF 426 and CPBF 427. Red lines delineate the applied threshold of 40% identity and 75% query coverage. Best blast results and accession numbers of sequences used as query are disclosed in Table S3.
